# Living Lab Dementia: Mixed-methods process evaluation of a feasibility study of an academic-practice partnership in German long-term dementia care

**DOI:** 10.1186/s12877-026-07164-9

**Published:** 2026-02-20

**Authors:** Felix Bühler, Andrea Leinen, Anja Bieber, Sascha Köpke, Gabriele Meyer, Swantje Seismann-Petersen, Martin N. Dichter

**Affiliations:** 1 Institute of Health, Midwifery and Nursing Science, Medical Faculty of the Martin Luther University Halle-Wittenberg, University Medicine Halle, Magdeburger Straße 8, Halle (Saale), 06112 Germany; 2 Institute of Nursing Science, Faculty of Medicine and University Hospital, University of Cologne, Gleueler Straße 176-178, Cologne, 50935 Germany

**Keywords:** Living lab, Academic-practice partnership, Long-term care, Dementia care, Patient and public involvement, Co-creation, Logic model, Process evaluation, Complex intervention

## Abstract

**Background:**

Structured partnerships between academia and nursing practice are likely to promote evidence-based practice and the involvement of healthcare professionals and patients in research. However, systematic evaluations of these partnerships are lacking. Therefore, we adapted the *Limburg Living Lab*, an academic-practice partnership, and carried out a feasibility study in German long-term dementia care. The three components of the *Living Lab Dementia* are *Linking Pins* (dyads of care professionals and researchers), facility-specific teams, and research teams. In this process evaluation, we examined the degree of implementation, the mechanisms of impact, and implementation barriers and facilitators.

**Methods:**

This convergent mixed-methods process evaluation was based on recommendations from the UK Medical Research Council framework and guided by a logic model. Quantitative data were collected via questionnaires and process documents (*n* = 195) and analysed descriptively. Qualitative data were gathered through individual interviews and focus groups with participants of the Living Lab Dementia (*n* = 32) and analysed using content analysis. Data were integrated by merging and comparing the two data sets.

**Results:**

Facility-specific teams and Linking Pins were implemented in four care facilities. Supported by facility staff, they identified research topics and carried out joint research projects to generate new knowledge on dementia care. People with dementia advised these projects through an external working group. The Linking Pins were involved substantially in all Living Lab activities, but perceived their roles as being demanding, given their numerous responsibilities. Implementation barriers included cultural differences between research and practice, and staff turnover. Facilitators were related to interpersonal relationships and structured exchange formats.

**Conclusions:**

This study concludes that collaboration between care professionals and researchers in a Living Lab is feasible, and joint research projects are an important mechanism for knowledge circulation. The Linking Pins require thorough role preparation to fulfil the numerous requirements and to involve all interest-holders. Facility-specific teams can be a valuable resource for involving care professionals in joint projects. These findings provide a foundation for future implementation efforts. Further research might focus on Living Lab outcomes and explore the role of the research team, as we were unable to investigate this component.

**Supplementary Information:**

The online version contains supplementary material available at 10.1186/s12877-026-07164-9.

## Background

Evidence-based practice is widely acknowledged as the key to providing quality care by integrating the best available evidence into clinical decisions [1]. However, the translation of research findings into care practice is slow [2], and evidence-based nursing is still not widely implemented [3].

Partnerships between academia and nursing practice are recognised as a promising approach to promote evidence-based practice in health care and to integrate research into practice by involving clinical staff in research activities [4]. Following calls from the American Association of Colleges of Nursing (AACN) [5, 6] for increased collaboration between nursing practice and research, numerous academic-practice partnerships have been established. These partnerships have been used to promote staff education [7], knowledge translation [8], or joint knowledge production and research [9]. However, systematic evaluations of academic-practice partnerships are lacking [10].

The Limburg Living Lab in Ageing and Long-Term Care, developed at Maastricht University (Netherlands), is one example of a long-standing academic-practice partnership. Within the Living Lab, healthcare professionals, researchers and older people collaborate on joint research projects aiming to improve long-term care [11]. While the Limburg Living Lab has produced considerable scientific, practical and societal impact in the Netherlands over the past 25 years [11] and has since been adopted in various European countries [12, 13], it has not yet been systematically evaluated.

**Fig. 1:**
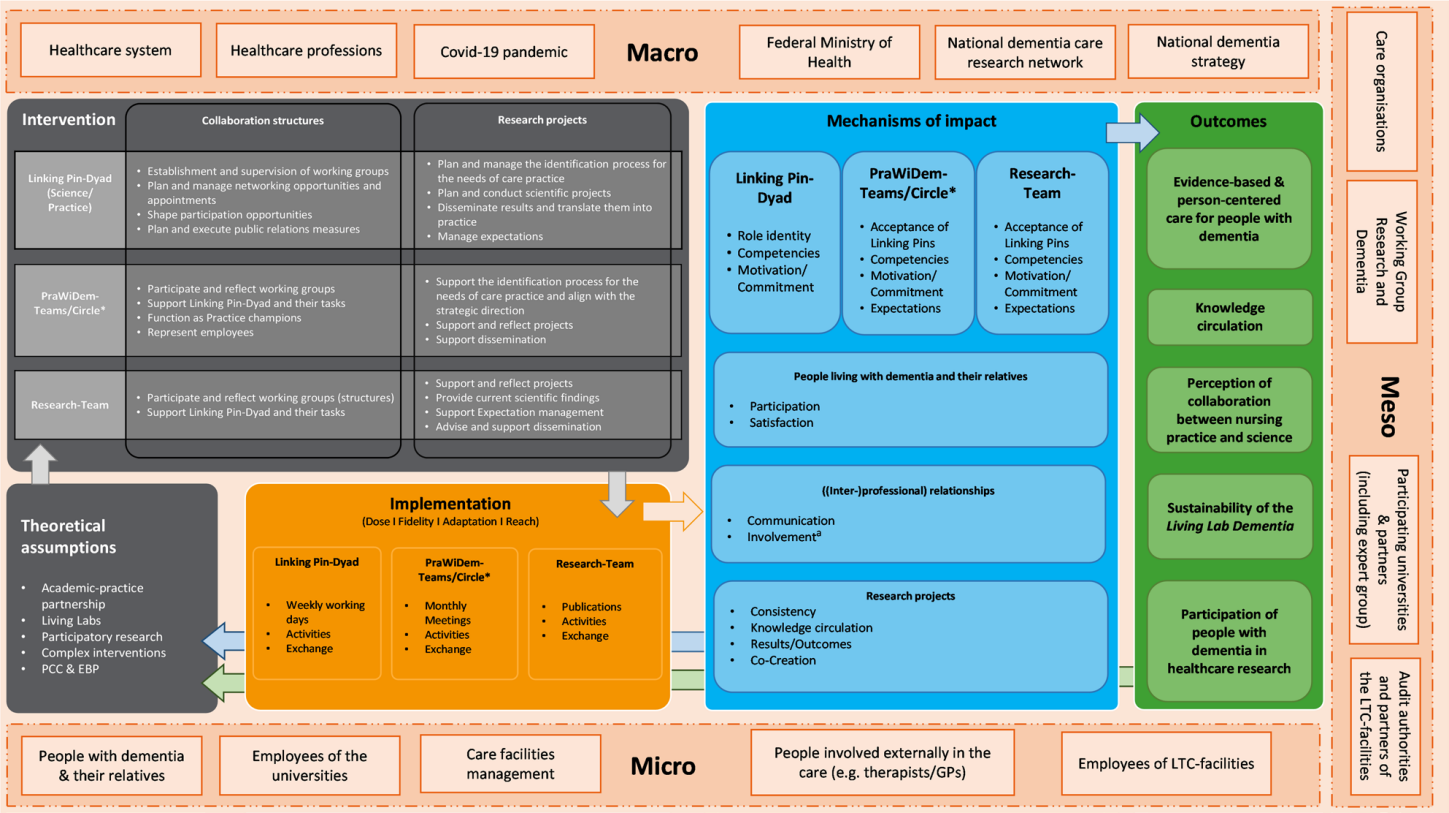
Logic model of the Living Lab Dementia (originally published in [18]). ^a^In order to correct a translation error in the original version [18], we changed the term “co-creation” to “involvement”. *EBP* Evidence-based practice *PCC* Person-centred care, *GP* General practitioner

Therefore, we aimed to adapt, implement and evaluate the Living Lab approach as a complex intervention in long-term dementia care in Germany. Guided by the UK Medical Research Council (MRC) framework [14], we have adapted the Living Lab approach based on consultations with our key interest-holders [15], preliminary findings from a systematic scoping review [16], and recurring expert consultations with various employees of the Limburg Living Lab. We developed a programme theory describing the research-focused academic-practice partnership [17] ‘Living Lab Dementia’ and visualised it in a logic model (Fig. 1 [18]). As part of the PraWiDem study (German acronym for *linking professional nursing practice and research in dementia*), we conducted this feasibility study of the Living Lab Dementia in Germany.

Alongside the 18-month implementation period, we carried out a mixed-methods process evaluation of the Living Lab Dementia. As suggested for the feasibility assessment of complex interventions [14], this evaluation focused on understanding the implementation process and its context, and the change mechanisms within the intervention – a recommended step before proceeding to the evaluation of intervention outcomes, i.e. the next phase of the MRC framework. Accordingly, our process evaluation aimed to investigate the feasibility of the Living Lab Dementia by describing (i) the degree of implementation, (ii) the mechanisms of impact, as well as (iii) the implementation barriers and facilitators. An evaluation of intervention outcomes was not conducted.

## Methods

The study design was guided by the UK MRC framework [14, 19]. Ethical approval was obtained from the ethics committee of the German Society of Nursing Science (no. 22–035) and a detailed study protocol for the process evaluation has been published [18]. In order to gain a thorough understanding of the intervention, we chose a convergent mixed-methods design [20]. The study was conducted at two sites in eastern and western Germany, each involving a university-based research institute and two long-term care (LTC) practice partners. In Saxony/Saxony-Anhalt, these included a small-scale dementia care facility and a home care service. In North Rhine-Westphalia, both facilities used an integrated neighbourhood-based care model, where staff provided nursing care for residents living in the care facilities, but also outpatient care for people living in the neighbourhood. Each of the four facilities was interpreted as one cluster Fig. 1.

### Description of the intervention

The complex intervention Living Lab Dementia consists of three intervention components, with each component acting on two levels, i.e. collaboration structures and research projects. A detailed description of the intervention is provided in our study protocol [18]:

1. The Linking Pin (LP) dyad consists of a practice-based LP (care professional) and a scientific LP (researcher). The LPs collaborate through weekly joint appointments to establish collaboration structures between the care facilities and universities. They initiate and carry out co-creative research projects within their respective care facilities and lead their facility’s multidisciplinary team.
2. The PraWiDem team members are employees of the care facilities who support the establishment of the Living Lab Dementia. They collaborate in monthly meetings and assist the LPs in identifying topics for research projects, implementing them and disseminating the results. PraWiDem team members have different professional backgrounds or positions; residents, relatives or other interested parties may join the teams. The involvement of management staff is crucial for the Living Lab Dementia; therefore, management staff from each cluster are involved at the team level. If multiple care facilities from the same organisation participate in the Living Lab, management involvement occurs at the level of the PraWiDem circle – an additional structure which enables cross-facility exchange through quarterly meetings and includes mid- and senior-level leaders of the organisation and the universities.
3. The research team consists of researchers from the participating universities who support the activities of the LPs and PraWiDem team members, provide current scientific knowledge and supervise the research projects. Research team members have different academic qualifications and professional backgrounds in healthcare.

### Sampling and data collection

A criteria-based convenience sampling strategy was applied and recruitment was mainly planned through direct approaches, flyers and advertisements. The participants recruited for the implementation of the intervention components were the same as for the data collection of this process evaluation. As recommended for a convergent mixed-methods design [20, 21], identical sampling was applied to quantitative and qualitative data. Contrary to our original study protocol, the intervention component “research team” was only considered for sociodemographic data collection, but was excluded from all other data collections due to overlapping roles of some participants as research team members and scientific LPs. Additionally, the perspectives of the ‘Dementia and Research’ working group were taken into account, a working group of people with dementia founded in collaboration with the German Alzheimer Association, who supported the development of the research projects carried out in the care facilities.

Data were collected before the start of the intervention (T0, July to August 2022), continuously during the 18-month intervention phase (ongoing, September 2022 to February 2024), and at the end of the intervention phase (T1, March to April 2024). The logic model [18] guided the data collection. The degree of implementation was assessed on the cluster-level, based on the four dimensions of the MRC framework: dose, reach, fidelity and adaptations [19]. All data collection methods, target groups and time points are displayed in Table 1.

**Table 1:** Overview of data collection

Domain and dimension	Information analysed	Target group	Data collection method(s)	Data type	Measurement point^a^
Implementation: dose	Number of intervention components and participants (overall and within clusters) Number of exchanges within components Number of public relations measures	n.a.	Document analyses	Quantitative	Ongoing
Implementation: reach	Characteristics of participants Reasons for (non-)participation Recruitment processes	LPs PraWiDem teams/circle CEOs Research team	Questionnaire (1) Document analyses	Quantitative	T0 + ongoing
	Reasons for participation of care facility	CEOs	Individual interviews	Qualitative	T1
Implementation: fidelity^b^	Extent of implementation of intervention components	n.a.	Document analyses	Quantitative	Ongoing + T1
	Support requirements of LPs Project planning processes	LPs PraWiDem teams/circle Nursing management staff	Individual interviews Focus groups	Qualitative	T1
Implementation: adaptation	Adaptation processes and reasons for adaptations	LPs	Individual interviews	Qualitative	T1
Mechanisms of impact: intervention components	Role identity, competencies, motivation/commitment of LP dyad	LPs	Individual Interviews	Qualitative	T1
	Acceptance of LP role, competencies, motivation/commitment and expectations of teams/circle	PraWiDem teams/circle Nursing management staff	Focus groups		
Mechanisms of impact: people with dementia and their relatives	Involvement opportunities for people with dementia and their relatives (if applicable) Satisfaction with involvement opportunities	LPs Working group ‘Dementia and Research’	Individual interviews Focus group	Qualitative	T1
Mechanisms of impact: (inter-)professional relationships	Communication and participation	LPs PraWiDem teams/circle Nursing management staff	Individual Interviews Focus groups	Qualitative	T1
Mechanisms of impact: research projects	Consistency, knowledge circulation, outcomes, co-creation	LPs PraWiDem teams/circle	Document analyses Individual interviews Focus groups	Quantitative + Qualitative	Ongoing + T1
Contextual factors	Factors facilitating or hindering the implementation of intervention components at micro-, meso- or macro-levels	LPs PraWiDem teams/circle Nursing management staff CEOs	Individual interviews Focus groups	Qualitative	T1
	Characteristics of participating care facilities	n.a.	Questionnaire (2)	Quantitative	T0

*n.a.* not applicable, *LPs* Linking Pins, *CEOs* Chief Executive Officers

^a^ T0 = baseline data collection before the start of the intervention, T1 = after 18 months, ongoing = continuous data collection during 18 month intervention phase

^b^ To assess the implementation fidelity (“Was the intervention implemented as planned?”), information on the LPs’ planning activities were collected

Qualitative data included individual interviews and focus groups, quantitative data were collected using questionnaires and process documents. The LPs documented all Living Lab activities (appointments, recruitment efforts, exchange with interest-holders, consultations with the working group ‘Dementia and Research’) in protocols and process documents. Process documents comprised logs of weekly LP activities and records of recruitment efforts for the PraWiDem teams and circle. Protocols included attendance lists and content summaries of all regular working meetings (weekly LP appointments, team and circle meetings), and summaries of discussions and consultations with any other interest-holders (staff, residents, CEOs, working group ‘Dementia and Research’). These documents were later used to collect data on the degree of implementation, LP and team activities, and to describe the co-creation within the care facility-specific research projects. Participants’ sociodemographic characteristics and the care facilities’ characteristics were collected with self-designed questionnaires at baseline.

Qualitative data were collected via semi-structured individual interviews (LPs and CEOs) and focus groups (PraWiDem teams, nursing management staff, working group ‘Dementia and Research’). Interview guidelines (provided in Supplement 1) were developed for each target group and tested in advance with healthcare staff not engaged in the study (e.g. nurses, occupational therapists). The interviews and focus groups were conducted by post-doctoral researchers or PhD students, some of whom held positions as scientific LPs. In view of this dual role and its potential impact on confidentiality and openness of discussions, interviewing researchers were exchanged between the participating care facilities, i.e. no scientific LP interviewed staff within their care facility. The scientific LPs themselves were interviewed by a researcher who had not been involved in previous Living Lab Dementia activities. Still, most interview participants were familiar with the interviewers and had previously met during Living Lab Dementia activities.

### Data analysis

Sociodemographic data were analysed descriptively, calculating frequencies, percentages and means using SPSS 28. To quantify the degree to which the presumed tasks were taken on by LPs and teams, we developed a code system based on the expected tasks defined in the logic model. Using the MAXQDA 2022 software, three researchers independently coded all LP and PraWiDem team protocols to identify which of the expected tasks the LP and team activities corresponded to. The results were subsequently discussed and summarised according to the logic model to display all LP and PraWiDem team activities carried out during the implementation phase. Similarly, LP and team protocols were analysed to quantify the degree of co-creation within each care facility’s research project. Co-creation was defined as the involvement of either care facility staff, residents with dementia, or residents’ relatives in the development and realisation of the research projects. For each of these three interest groups, we re-coded the LP and PraWiDem team protocols with a four-code system based on the four phases of the collaborative process: co-ideation (i), co-design (ii), co-implementation (iii) and co-evaluation (iv) [22]. The involvement of practice-based LPs was not considered for this analysis, as we expected them to be part of the majority of Living Lab activities, hence analysing their involvement would have biased our analysis towards a higher degree of co-creation.

Interviews and focus groups were audio recorded, transcribed verbatim and analysed using a structuring content analysis [23]. We developed a code system based on the research questions, with subcodes according to the domains of this process evaluation as described in Table 1. All interview data were coded deductively with this code system (provided in Supplement 2). Coding was performed by three of the researchers who conducted the interviews. As in the previous steps, through exchange between the study centres, it was ensured that the scientific LPs did not analyse interviews from their collaborating care facilities. All coded transcripts were exchanged between study centres and mutually cross-checked for consistent application of the code system; discrepancies were resolved through discussions between the researchers. After coding, the data were first summarised across interview and code levels, and then further condensed into code summaries, which were exchanged equally between study centres and cross-checked between researchers. Given our objective of describing implementation barriers and facilitators at the contextual level, we sought to map our findings onto an internationally recognised framework for implementation determinants [24]. Consequently, we structured our summaries of implementation barriers and facilitators along the domains of the updated Consolidated Framework for Implementation Research (CFIR) [25], which is among the most widely used and well-established determinant frameworks in implementation research [26, 27].

We analysed quantitative and qualitative data separately in accordance with the convergent mixed-methods approach [20]. The results-based integration was performed by comparison of the two data sets, and by discussing and interpreting their alignments, differences and complementarity.

## Results

### Sample and recruitment

In total, 44 participants from three universities and four care facilities were recruited. The care facilities were recruited through the researchers’ professional networks. Characteristics of the facilities and information on the research projects carried out within the Living Lab are displayed in Table 2. All four research projects addressed topics identified within the care facilities and related to the needs of people with dementia receiving nursing care. The main focus of these projects was to co-create knowledge for future use in dementia care, e.g. how care staff may promote independence for people with dementia, or whether communication with people with dementia may be supported through the use of pictograms.

**Table 2:** Characteristics of participating care facilities and research projects within the Living Lab Dementia

Cluster	01	02	03	04
**Care facility characteristics**				
Nursing services (max. number of patients)	Inpatient care (91) Home care (70) Day care (14)	Inpatient care (30)	Inpatient care (84) Outpatient care (11) Assisted living (27)	Inpatient care (90) Outpatient care (42) Assisted living (42)
Residential concept for inpatient care	Housing community and traditional care units	Housing community	Housing community	Traditional care units
Nursing focus	-	Dementia	Integrated neighbourhood-based care	Integrated neighbourhood-based care
Staff (number)	Skilled nurses (34) Nursing aides (30) Social care assistants (16)	Skilled nurses (8) Occupational therapist (1) Nursing aides (7) Social care assistants (3)	Skilled nurses (16) Nursing aides (27) Social care assistants (16)	Skilled nurses (19) Nursing aides (28) Social care assistants (10)
Dementia-specific care concepts	-	Specialised psychogeriatric care in a dementia-friendly building, all residents have dementia	-	Open psychogeriatric wards
**Research projects**				
Research topic	Promoting self-determination and independence in people with dementia	Sensory stimulation for immobile residents with dementia	Supporting the communication of daily needs and wishes of people with dementia (“ComDem”)	Managing food and fluid refusal in the care of people with dementia (“Autonomy in Nutrition”)
Aims	• Enabling employees to recognise and take into account opportunities for self-determination and independence for people with dementia in care • Development of a guide with practical recommendations to promote self-determination and independence	• Development and feasibility testing of interventions for sensory stimulation for immobile residents with dementia • Implementation of feasible stimulation interventions at the level of the entire care organisation	• Development and feasibility testing of pictograms to support people with dementia in expressing their preferences regarding breakfast and dinner choices as well as the selection of daily activities • Development of a training program to facilitate the intervention across all facilities by the care provider	• Development of a literature-based questionnaire to assess the current state of practice and challenges encountered by care staff when dealing with residents’ refusal of food and drink. • Cross-sectional survey of all care staff in the care organisation • Results-based recommendation of a target-group specific intervention
Methods	Knowledge-to-action-process	Knowledge-to-action-process	Mixed-methods feasibility study	Cross-sectional study
Project status at the end of the 18-month implementation phase	• A model of enhancing self-determination and independence in dementia was developed • Translation and implementation of the model in daily care practices were started	• Six interventions were developed, tested and evaluated within the participating care facility, but not yet implemented at the level of the care organisation.	• Seven staff training sessions and data collection activities (individual interviews, focus groups, and documentation protocols) were completed, and data analysis was ongoing.	• A validated questionnaire was developed and data were collected and analysed (*n* = 132); results were presented and interprofessional reflection rounds were ongoing to identify the intervention

We collected sociodemographic data from 41 Living Lab participants and four members of the ‘Dementia and Research’ working group. Documentation analysis was carried out using process documents and protocols from the weekly LP activities (*n* = 158), protocols from monthly PraWiDem team meetings (*n* = 32), documentation of the recruitment processes within the clusters (*n* = 4), and documentation of cross-cluster Living Lab activities (*n* = 1). Qualitative data were collected via individual interviews with LPs (*n* = 7; mean duration 87 min, range 66–118 min) and CEOs (*n* = 3; mean duration 34 min, range 22–41 min). Focus groups were conducted with the PraWiDem teams in each of the four clusters (*n* = 14 participants in total; mean duration 58 min, range 54–66 min), and additionally for nursing management staff across the clusters (*n* = 5 participants; 68 min). One PraWiDem team member was unable to attend the focus group and took part in an individual interview (54 min) as their perspective was considered highly relevant to this process evaluation due to their responsibility for projects within the care facility. Finally, an online focus group was carried out with the ‘Dementia and Research’ working group (*n* = 2 participants; 55 min).

We present our findings according to the elements of our logic model. As we expected the intervention to affect LPs and team members differently, we formulated different mechanisms of impact for these target groups. Accordingly, we investigated the LPs’ and PraWiDem teams’ degree of implementation as intervention components, and additionally analysed the mechanisms of impact found at their respective levels. For more clarity, we present our findings on the implementation and mechanisms of impact separately for the LP dyads and PraWiDem teams. In view of our small sample size, illustrating quotations are presented only with the participants’ role within the Living Lab, but without their cluster affiliation in order to ensure privacy.

### LP dyad

#### Implementation

**Fig. 2:**
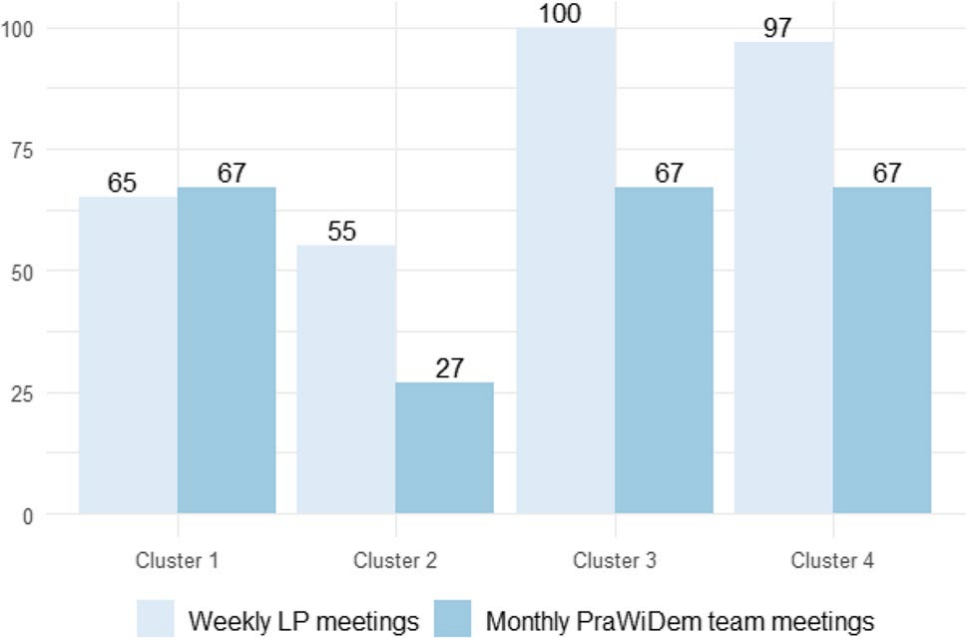
Delivered doses of Linking Pin meetings and PraWiDem team meetings in %. Based on public holidays and annual leave of employees in Germany, we assumed 44 working weeks per year, i.e. 66 weekly Linking Pin (LP) meetings during the 18-month implementation phase. Accordingly, we assumed ten PraWiDem team meetings per year, i.e. 15 meetings during the implementation phase

Eight participants were recruited to the LP roles. The dyads were implemented in all clusters, but prolonged sick-leave and staff turnover led to changes in the LP roles in two clusters. Originally, there were three scientific LPs (SLPs), one of whom was responsible for two care facilities from the same organisation (clusters 3 and 4). However, due to a period of prolonged illness, this SLP was temporarily replaced by a colleague. The delivered doses of weekly LP meetings varied between 55% and 100% (Fig. 2).

The practice-based LPs (PLPs) were recruited by management staff (clusters 1 & 2) or internal job advertisements (clusters 3 & 4). All PLPs worked in direct patient care and held additional management positions with personnel responsibility, one PLP held a bachelor’s degree in nursing science. The SLPs were recruited from the universities and had different levels of research experience. Details on the sociodemographic characteristics of the LPs are displayed in Table 3.

**Table 3:** Participants’ characteristics

Living Lab Dementia participants				
	Age, years range (mean)	Gender *n* (%)	Professional background *n* (%)	University level education *n* (%)
Practice-based LPs *n* = 4	24–38 (29.0)	F: 4 (100) M: 0	Nursing: *n* = 3 (75) Occupational therapy: *n* = 1 (25)	Bachelor level; *n* = 1 (25) None: *n* = 3 (75)
Scientific LPs *n* = 4	27–53 (35.5)	F: 3 (75) M: 1 (25)	Nursing: *n* = 4 (100)	Master level: *n* = 3 (75) PhD level: *n* = 1 (25)
PraWiDem team members *n* = 22	24–72 (45.1)	F: 18 (81.8) M: 3 (13.6) Missing: 1 (4.5)	Nursing: *n* = 10 (45) Nursing assistance: *n* = 4 (18) Social care assistance: *n* = 2 (9) Housekeeping: *n* = 2 (9) Occupational therapy: *n* = 1 (4.5) Social work: *n* = 1 (4.5) Not specified: *n* = 2 (9)	Diploma:^a^ *n* = 1 (4.5) Master level: *n* = 2 (9) Not specified: *n* = 3 (13.5) None: *n* = 16 (72.7)
Nursing management staff and care facility CEOs *n* = 6	32–62 (52.5)	F: 5 (83.3) M: 1 (16.7)	Nursing: *n* = 3 (50) Management: *n* = 2 (33.3) Housekeeping: *n* = 1 (16.7)	Bachelor level *n* = 1 (16.7) Not specified *n* = 1 (16.7) None: *n* = 4 (66.7)
Research team *n* = 5 (without SLPs)	43–64 (55.4)	F: 1 (20) M: 4 (80)	Nursing research: *n* = 3 (60) Medicine *n* = 2 (40)	State examination:^b^ *n* = 1 (20) PhD level: *n* = 1 (20) Professor: *n* = 3 (60)
‘Dementia and Research’ working group				
	Age range (mean)	Sex n (%)	Current housing situation and support n (%)	Years since diagnosis range (mean)
‘Dementia and Research’ working group members *n* = 4	55–78 (65.3)	F: 2 (50) M: 2 (50)	Living with relatives, no support: *n* = 1 (25) Living with relatives, support with chores: *n* = 2 (50) Living alone, support with chores and medication: *n* = 1 (25)	1–4 (3)

*LPs* Linking Pins, *F* female, *M* male

^a^ Before the introduction of Bachelor’s and Master’s degree programmes, diploma programmes with a study duration of 4 years were common in Germany

^b^ Study duration 6 years

Regarding implementation fidelity, our documentation analysis revealed that the LPs carried out the majority of the activities presumed in the logic model, with only a few exceptions, e.g. dissemination of results (Table 4). However, our interviews indicated that this was due to the fact that the joint research projects within the Living Lab Dementia had not been finalised at the time of data collection (T1). The LPs stated that the Living Lab collaboration involved a lot of planning, e.g. preparation and follow-up of meetings, LP tasks and appointments. Especially early on, a lot of the planning activities were carried out by the SLPs. While the LPs were generally familiar with the central Living Lab tasks at the beginning of the collaboration, there were only few organisational specifications for implementation beside the intended exchange formats. This led the LPs to develop their own routines and collaboration structures in each cluster, e.g. fixed daily routines for LP appointments or regulations on dealing with documentation. The frequency and duration of planned exchange were handled flexibly and adjusted regularly (shortened or online meetings), e.g. in cases of care practice responsibilities colliding with LP appointments, or if LPs felt there was no need for exchange.

The PLPs reported that they required support from the SLPs and research teams to work on the research projects, especially regarding scientific tasks (collecting data, writing and presenting proposals etc.) and acquiring computer and software skills. Nursing managers supported the PLPs by providing organisational support, time and technical resources (hardware etc.), and also displayed support publicly to underscore the importance of the new role. *“Whenever I saw the need to support the two of them in a staff meeting*,* I did so. You always have to encourage the staff. That is where the two [LPs] need support.”* (nursing manager).

The SLPs’ support needs mostly related to developing role security for their new role and to planning research projects that could be carried out collaboratively with all interest-holders. The less experienced SLPs in particular reported that as ‘novice researchers’ they needed support from experienced researchers. The SLPs therefore held frequent consultations with their research team, each other, or with the SLPs from the Limburg Living Lab, to reflect on the SLP role. “*This was my first project after completing my master’s degree. […] It was very important for me to have someone who gave me confidence*,* especially in the beginning.”* (SLP).

In summary, the implementation of the LP dyad can be considered successful. Implementation challenges arose from longer absences of staff in LP roles and frequent collisions between LP appointments and practice responsibilities of PLPs, especially in clusters 1 and 2. However, the LP exchange and activities were mostly carried out as planned, and the LPs took on the majority of the tasks outlined in the logic model Table 4.

**Table 4:** Observed LP and PraWiDem team activities

	Activities predefined in the logic model	Observed activities
LPs: Collaboration on research projects	Identify care practice needs as topics for the research projects	• Communicate with all interest-holder groups (e.g. staff, management, works council) • Involve staff, residents and other interest-holders in topic identification process, e.g. through World Cafés, informal conversations, notice boards • Collect, analyse and communicate collected proposals
	Plan and conduct research projects	• Draft, communicate and refine project plan with interest-holders • Carry out project tasks and coordinate them with the project plan • Research and consider external evidence and research methodology • Obtain ethical approval • Collect and analyse data • Develop and test interventions with involvement of care facility staff
	Translate and disseminate findings	-
	Manage expectations	• Communicate with management staff • Manage expectations between the LPs
LPs: Establishment of collaboration structures	Work organisation	• Acquire necessary software and hardware • Plan, reflect and refine working structures in collaboration with interest-holders (management staff, quality management etc.) • Deal with staff changes and absences • Manage appointments • Learn and apply project management methods
	Plan and execute public relations measures	• Homepages • Social media • Regional and facility newspapers • Flyers • Participation incentives • Posters • Plan, conduct and evaluate public information events
	Shape participation opportunities	• Obtain informal feedback (shift handover, nursing staff meetings, informal conversations with (management) staff, residents, other interest-holders) • Plan and conduct workshops with staff, residents, relatives
	Plan and manage networking opportunities and appointments	• Networking within the care facility (e.g. with staff and residents not directly involved in the Living Lab Dementia) • Networking within the care organisation (with staff and residents not directly involved in the Living Lab Dementia), e.g. employee representative council • Networking with external interest-holders (e.g. general practitioners, therapists, palliative care) • Cross-cluster networking (other facilities and organisations within the Living Lab Dementia) • International networking (with other Living Labs) • Conferences
	Establish and supervise working groups	• Manage appointments and book rooms for meetings • Draft presentations and materials • Document meetings
PraWiDem team members: Collaboration on research projects	Support dissemination	-
	Support and reflect research projects	• Provide care practice perspective to advise the research projects • Identify involvement opportunities and support interest-holder involvement • Reflect progress of research projects • Support recruitment and data collection • Take on role of multipliers within care facility (e.g. to involve facility staff or external interest-holders, drawing attention to current project steps and tasks requiring staff support) • Support intervention development • Support public relations measures • Support in preparing result presentations • Integrate and systematically reflect on research project topic in one’s own work
	Support identification of care practice needs as topics for the research projects and align with the strategic direction of the care organisation	• Propose and prioritise topics for research projects • Align topics with organisational goals
PraWiDem team members: Establishment of collaboration structures	Work organisation	• Manage appointments • Plan and reflect working structures • Manage networking opportunities
	Represent employees	• Represent staff at cross-cluster Living Lab Meetings
	Function as practice champion	-
	Support LP dyad and their tasks	• Recruit PraWiDem team members • Support management of appointments • Provide facility and context specific information relevant to the establishment of working structures
	Participate in and reflect working groups	-

*LPs* Linking Pins

#### Mechanisms of impact

The LPs were for the most part motivated and curious to take on their new roles, but felt that while the tasks associated with their roles were clear, they were unsure how to carry out some of these tasks (e.g. how to involve staff in identifying a research topic). The PLPs received no particular role preparation. The SLP role, on the other hand, was taken on by researchers who had previously developed this role, for example through exchanges with SLPs from the Limburg Living Lab. While the initial role uncertainty was perceived as challenging, both PLPs and SLPs reported an increase in role security during the course of the collaboration, mainly through defining and subsequently accomplishing LP tasks, participating in cross-cluster Living Lab meetings and (national or international) exchange with other LPs.

The PLPs considered themselves to be the practical part of the LP dyad and emphasised the need to be well established in nursing practice in order to take on their role. *“You definitely need practical experience and contact with staff.”* (PLP). They had little prior knowledge of research methods. Some PLPs reported that the collaboration increased their research knowledge and skills, but also provided them with other new skills, e.g. giving presentations or learning IT skills. The PLPs indicated many skills relevant to their role, e.g. being able to involve other care professionals in the Living Lab, or the ability to work persistently on a longer project despite setbacks. While they considered the willingness and courage to learn new things to be important for their role, some PLPs found it difficult to assess what they had learned in retrospect.

The SLPs reported that they needed a profound understanding of the practice setting to fulfil their role and develop the participatory research projects. Being present in the care facilities during the weekly LP appointments and work shadowing were considered helpful for building relationships and creating the strong focus on practice needs required to understand the topics relevant to care professionals. In order to bring together the research and practice perspectives, the SLPs needed the ability to translate and effectively communicate research topics in practice terms. Despite the focus on practice needs, research skills were crucial for the SLP role. The inexperienced SLPs reported feeling less confident in planning the research projects and therefore sought more exchange with their research team or their academic supervisors. The SLP role was considered challenging and going well beyond the typical job profile of a researcher. *“It is a very demanding task. Which topic will be addressed*,* finding suitable methods to address it*,* having the right skills to be able to address it.”* (SLP).

In summary, the LPs were mostly motivated and committed to their tasks. However, they found their roles challenging, especially at the beginning. This was due to the initial role uncertainty and the numerous skills required for the LP roles, not all of which were available from the outset or could be acquired over the course of the collaboration.

### PraWiDem team/circle

#### Implementation

A total of 22 participants were recruited to the four PraWiDem teams, with team sizes between three and seven persons. The delivered dose of monthly PraWiDem team meetings was 67% in three clusters. In cluster 2, the dose was noticeably reduced (27%) (Fig. 2). Our interview data suggest that the speed at which the research projects progressed did not always warrant monthly team meetings. “*We always checked whether there was a need that month or whether there was no topic. While the survey data were being analysed*,* there simply were no topics during that time. So it sometimes happened*,* that nothing [no meetings] took place for two or three months*.” (PLP). The additional exchange structure, the PraWiDem circle (only in cluster 3 and 4, as they belonged to the same care organisation), comprised seven mid- and senior-level leaders from care organisations and universities. Additionally, the exchange format was open to interested managers from other care facilities within the organisation, as well as to staff from quality management and representatives of the works council. All six quarterly PraWiDem circle meetings took place as planned (100%).

PraWiDem team members were mostly recruited by the LPs, with potential participants identified by PLPs or management staff. In some clusters, the team composition reflected all professions involved in care, while the team in one cluster consisted of only nursing staff. No residents with dementia were recruited for the PraWiDem teams, and only one relative of a resident took on the team role. The members of the PraWiDem circle were selected for participation by the CEO of the organisation, based on their leadership roles and positions (e.g. head of nursing staff).

The majority of the PraWiDem team members had nursing backgrounds and no academic education. Most team members stated that they participated out of curiosity about the Living Lab approach or because of the opportunity for personal development. Sociodemographic characteristics of the PraWiDem team members and management staff participating in the PraWiDem circle are displayed in Table 3.

Documentation analyses and comparison with the logic model revealed that the team members mostly focused on the research projects, but carried out fewer tasks relating to the establishment of collaboration structures within the Living Lab (Table 4). This was mirrored by the focus groups with the PraWiDem teams, in which the team members reflected extensively on their involvement in the research projects, but hardly discussed the overall Living Lab structure and their role within it.

In summary, the implementation of the PraWiDem teams was not fully successful. The team component did not reach all of the intended target groups. Only some PraWiDem teams were multidisciplinary. The team members were mostly involved in the research projects as planned, but their involvement in the establishment of collaboration structures was low. While the frequency of PraWiDem team meetings was lower than planned, regular exchange with staff took place in all clusters, but often outside team meetings. In cluster 2, for instance, exchange with staff frequently occurred in official department meetings instead of PraWiDem team meetings. The PraWiDem circle proved to be a valuable structure for cross-facility exchange within one care organisation and was successfully implemented in terms of reach and dose.

#### Mechanisms of impact

The acceptance of the LP dyad was generally high; the PraWiDem team members perceived the LPs as team-oriented and approachable. *“I find her [SLP] very likeable. It is easy to strike up a conversation with her.”* (team member). However, the availability of the LPs seemed to vary between the clusters, with some team members wishing for more presence of the LPs on site. *“I didn’t really have any contact with the two of them. It was really just this one meeting every month.”* (PraWiDem team member).

PraWiDem team members reported that their interest in the research projects and the desire to improve care for people with dementia initially motivated them to join the teams, but their contributions were often limited to the monthly team meetings due to lack of time. Not all expectations of the team members were apparent, with expectations ranging from scepticism towards the collaboration to tangible and readily available long-term changes in dementia care. Team members generally described the lively exchange within the teams as rewarding and felt that it led them to broaden their perspectives and develop new ideas for dementia care, but not all their expectations were met, especially regarding the application of results. “*I sometimes felt like we were sitting together and thinking about things that would be wonderful for people [with dementia] but in the end won’t be feasible under current conditions.*” (team member).

The PraWiDem team members emphasised that they needed strong organisational, social and communicative skills to encourage and involve other staff in the research projects. Research skills or experience were not considered a requirement for the team role, but the ability to reflect on care practice, willingness to learn and innovative skills were highlighted. However, our interviews did not clarify whether the team members acquired these skills over the course of the collaboration or whether they already had them beforehand.

Management staff perceived the LPs as professional and committed to their role, and emphasised that the presence of the SLP had the potential to stimulate a learning atmosphere within the care facility. However, it was also noted that some SLPs needed to take on an even stronger mediating role towards care professionals to highlight the practical relevance and benefits of research. While some management staff shared the team members’ expectation of implementable results, others considered the acceptance and conceptual integration of the Living Lab as an indicator for success. “*I believe that success is measured by how naturally this work is perceived in our company. Just as there is a managing director or a board of directors*,* there is a Living Lab here that further develops the [care] work.”* (care facility CEO).

To summarise, the acceptance of the LPs was generally high and the PraWiDem team members reported a high level of motivation due to their identification with the research projects, but their involvement often did not extend beyond the monthly meetings. The expectations varied and some team members were unsure what to expect from the collaboration. Although the PraWiDem team members had specific ideas about the skills required for the team role, it remained unclear whether these skills were acquired over the course of the collaboration.

### People with dementia

Involving residents with dementia from the care facilities was perceived as challenging due to their mostly advanced dementia stages. Although no residents were recruited to the PraWiDem teams, LPs and team members reported various efforts to involve residents with dementia informally, e.g. through conversations about the research topics or a World Café designed to identify potential research topics. Several SLPs expressed regret that the involvement of residents with dementia was not achieved satisfactorily, and referred to their own lack of knowledge about involvement methods as a barrier. “*I’m not sure whether this was due to my beginner’s role*,* but I haven’t managed to gain a comprehensive overview of the methods available for involving people with dementia.*” (SLP).

The LPs consulted the ‘Dementia and Research’ working group to include the perspective of people with dementia in the research projects. Given the challenges in recruiting people with dementia to the PraWiDem teams, these consultations remained the only involvement of people with dementia in most clusters. The working group advised the LPs at various project stages, for example by discussing the research topics in terms of their relevance to people with dementia, or by offering advice on how to address sensitive dementia issues with residents and relatives. A representative from the German Alzheimer Association whom the participants knew beforehand moderated the online consultations. The LPs presented their research projects and sought feedback from the working group using example scenarios and short presentations. The consultations were perceived as valuable for the research projects. However, the LPs did not consider the working group members to be representative of the original target group of residents with dementia, as they did not have advanced dementia or high care needs (see Table 3).

The working group members experienced their involvement in the research projects positively. They were motivated by the prospect of contributing to the improvement of dementia care and experienced their involvement as impactful. “*I always felt like I could contribute a lot [to the projects]*.” (person with dementia). Working group members stated that during the first few meetings, a common approach for collaboration needed to be established. Initially, the LPs were perceived as too cautious and did not challenge the working group enough. Over the course of the collaboration, however, the LPs involved the working group more intensively and trusted members to contribute more. *“At the beginning*,* there was still a feeling that the researchers were a little afraid of overwhelming us.”* (person with dementia).

In summary, residents with dementia came into little contact with the intervention. Whether they were satisfied with their involvement opportunities remained unclear. The members of the working group ‘Dementia and Research’ supported the research projects through consultations at various stages. These consultations required finding a common approach to collaboration, which ultimately resulted in a high degree of satisfaction on the part of the working group members.

### (Inter-)professional relationships

Overall, communication within the Living Lab was appreciated and considered as respectful. The LPs reported being primarily responsible for ensuring communication. They utilised various communication formats and adapted them to the individual facilities, e.g. regular meetings with management staff, communication with employees through regular department meetings, PraWiDem team and circle meetings etc. This resulted in a high communication density and tied up a lot of resources for the LPs. While the team members and management staff mostly reported feeling well-informed, the LPs pointed out that finding ways to involve and communicate with staff with no particular Living Lab role was challenging. *“It was extremely difficult to find a gatekeeper for the survey. We sent emails and made phone calls*,* but we couldn’t find anyone. We simply didn’t receive any feedback.”* (SLP). Annual Living Lab meetings for the LPs, PraWiDem teams and management staff were organised to foster relationships and promote cross-cluster exchange. These meetings and the international Living Lab exchange were valued as important platforms for communication about the Living Lab structures and roles, but there was little cross-cluster exchange between the care facilities outside of these meetings.

Collaboration was generally perceived as valuable, but required finding a common working approach. Especially early on, the SLPs reported that they had to take the lead, both within the LP dyad and in collaboration with the PraWiDem teams (e.g. preparing weekly LP appointments, keeping an overview of the LP and team tasks, drafting project proposals, instructing PLPs to take on tasks by themselves etc.). To enable collaboration as partners, it was necessary to achieve a balance between research and practice, e.g. jointly reading research articles and then discussing their relevance and implications for the research projects. For many care professionals, active long-term collaboration in a project was a new experience. *“There are no working groups in our organisation in which staff can actively collaborate on a topic and help develop it.”* (PLP). Consequently, the LPs reflected that care professionals were not used to a high degree of autonomy when it came to designing the research projects. Therefore, opportunities with specific tasks and a clearly defined scope of action were considered suitable for involving care professionals. These opportunities were mostly initiated by the SLPs.

In summary, the communication was largely initiated and organised by the LPs and required a lot of resources given the numerous communication formats within the Living Lab. To collaborate within the Living Lab Dementia, PraWiDem team members and LPs needed to establish a common working approach.

### Research projects

Challenges securing LP appointments were present in all clusters, e.g. staff changes, prolonged sick-leave of LPs or staff shortages forcing the PLPs to skip their LP appointments. These challenges were perceived to have a negative impact on the continuity of work on the research projects.

PLPs and PraWiDem team members reported that working on the research projects encouraged them to reflect and adapt their own care practice and communication. According to our interviews, knowledge circulation and exposure to new knowledge occurred through a variety of mechanisms within the Living Lab, e.g. collaborating on the research projects, participating in cross-cluster exchange or attending events such as conferences or presentations organised within the care facilities (e.g. a presentation by a neurologist regarding sensory perception in dementia). *“We have learnt a lot*,* also through the conferences we have attended. What is currently being worked on [in the field]? That’s very exciting*,* you don’t really get to see that in practice.”* (PLP). However, an increase regarding research-specific knowledge or skills was mostly experienced by the PLPs. PraWiDem team members, on the other hand, emphasised having gained increased awareness of dementia needs, but did not report an increase in research-specific knowledge.

Regarding the co-creative processes within the research projects, both our interview findings and documentation analysis showed that co-creation mainly occurred with staff from the care facilities. Exchange with staff who were not team members often occurred through informal conversations. People with dementia and relatives were involved only to a lesser extent. The results of the documentation analysis are displayed in Table 5. The comparison of our findings suggests that although care professionals and team members were involved in various stages and tasks of the research projects, they did not consider their involvement to be “scientific”, and emphasised that they had merely supported the LPs by contributing their practice perspectives or acting as multipliers. *“What we worked on here was hands-on. So what we were doing here didn’t really have much to do with science*,* did it? We were really only able to assist with our practical part.”* (PraWiDem team member). Still, the acceptance of the research projects among staff was considered high due to the co-creative development.

**Table 5:**
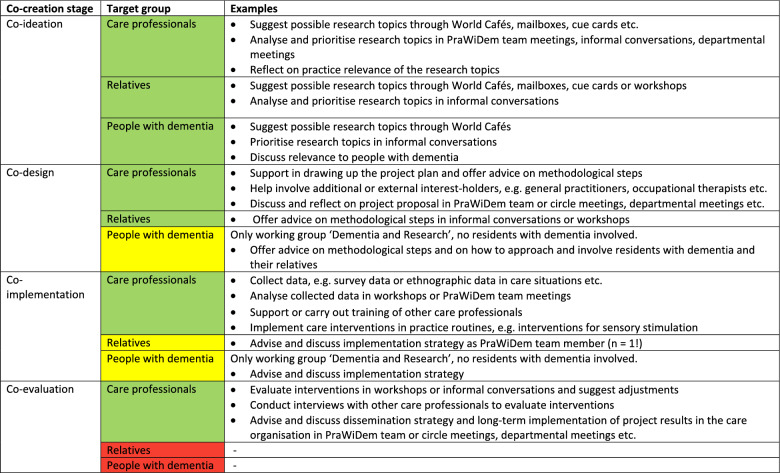
Co-creation within the research projects by target group

The colour gradient represents the level of co-creation: green = high, yellow = medium, red = none

Most results were not available at the end of the 18-month implementation phase as the research projects had not been completed. Some of the results that were available had already been utilised in care practice, which was judged positively by the PraWiDem team members (e.g. survey on dealing with food refusal in dementia, interventions for sensory stimulation). However, team members particularly emphasised that the results of the research projects were expected to demonstrate a tangible benefit for dementia care, which was not yet the case for all projects. Several PraWiDem team members expressed their wish that the implementation of the results in the care facilities should also take place within the Living Lab collaboration, guided by the LPs.

In summary, practice staff were co-creatively involved in all stages of the research projects despite challenges with continuity. Knowledge circulated through different mechanisms within the research projects. At the PraWiDem team level, knowledge circulation mostly related to the topics of the research projects, some LPs additionally reported increased knowledge regarding research methods and procedures. The projects’ results were largely expected to have a tangible impact in dementia care, which was not yet the case for all research projects.

### Barriers and facilitators

**Fig. 3:**
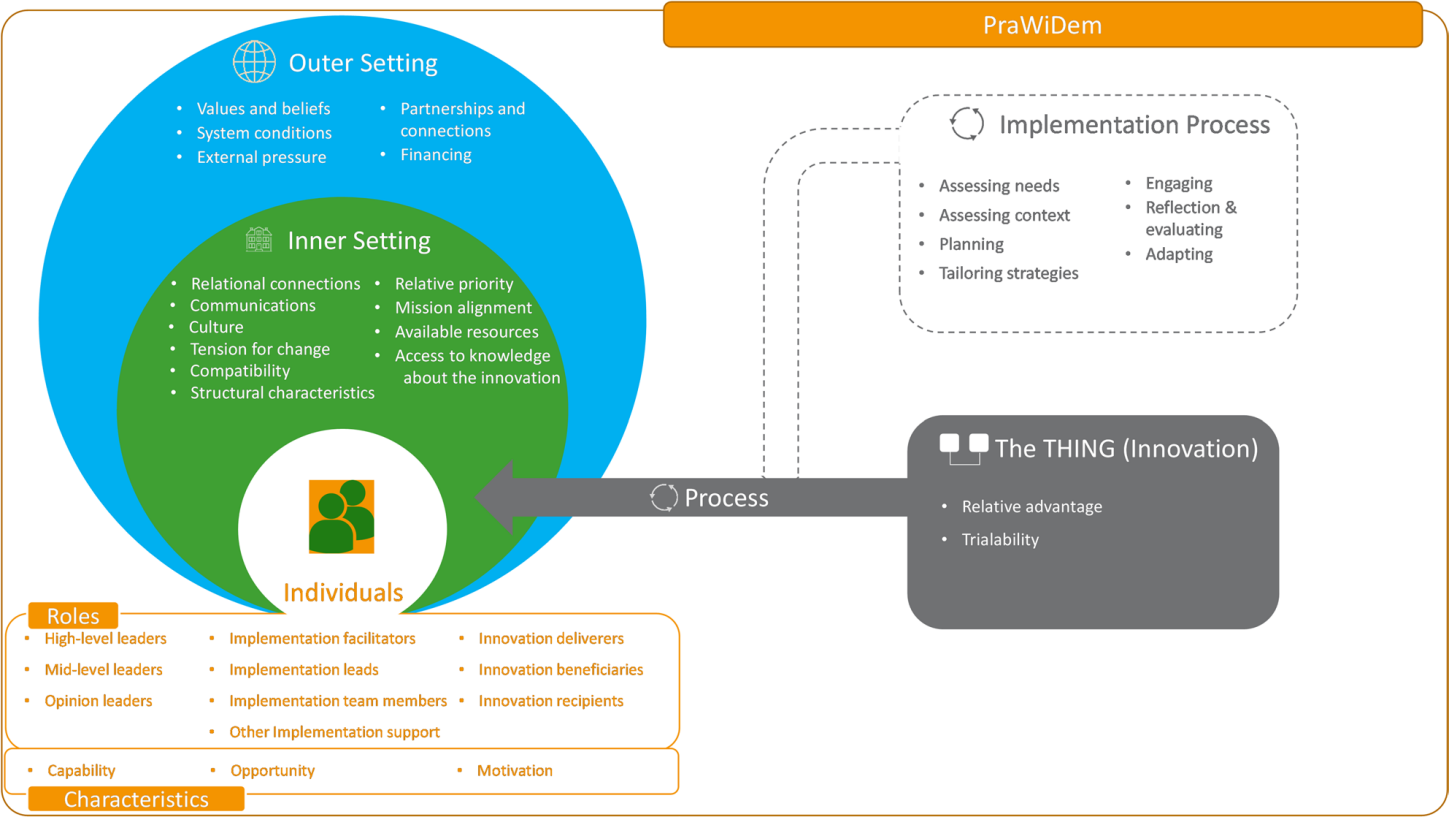
Adapted CFIR model with identified domains (based on the CFIR 2.0 [25], figure adapted by the Center for Implementation [28])

Figure 3 illustrates the specific subdomains in which implementation barriers and facilitators were identified.

Within the domain of Innovation, the jointly conducted research projects were perceived as an effective mechanism to activate and consolidate collaborative and network structures embedded in the Living Lab approach. Implementation was considered more feasible when initiated with a limited number of care facilities, each providing a clearly designated contact person. *“This was suggested to us by our colleagues in Maastricht*,* and I think it makes a lot of sense: ’Start small. Allow it to evolve*.” (SLP).

Within the Outer Setting, the prevailing perception that nursing staff primarily see themselves as hands-on practitioners rather than contributors to planning and evaluating research projects was a hindering factor, along with societal taboos around dementia and dying. On the other hand, it was beneficial that dementia as a topic was considered important by the institution and that there was a clear desire for further development. The cross-cluster exchange was perceived as supportive, also with regard to recognising the Living Lab not only at the level of jointly conducted research projects. External funding facilitated the initiation of the process, however, sustainable implementation of the Living Lab was only considered realistic, if long-term financial independence could be achieved. *“It was certainly also helpful that it was externally funded*,* which made it easier to say*,* ‘Okay*,* let’s give it a try.”* (care facility CEO).

In the Inner Setting barriers primarily reflect structural and organisational issues. Barriers included limited resources (e.g. lack of office space for the LPs, outdated equipment, poor internet access), long distance traveling for the scientific LPs and high workload (often caused by responsibilities outside the Living Lab). *“My practice-based LP was quite often distracted by day-to-day issues that had nothing to do with the research project.”* (SLP). Staff turnover (especially at LP or mid-leader level) and a lack of decision-making authority as well as challenges in reaching management staff and receiving timely feedback further hindered implementation of the Living Lab Dementia. Additionally, scepticism toward (nursing) science and a perceived lack of project-related value among some PraWiDem team members negatively influenced motivation and engagement. Facilitators included personal continuity and fixed and structured LP working days. *“What helps me is that we always start together. And even when I’m working on a task on my own*,* I have [SLP] by my side and can ask questions at any time.”* (PLP). Working with two PLPs (clusters 3 and 4) helped ensure continuity and bridge absences. Regular exchanges, informal or within PraWiDem team meetings (team and circle), and Living Lab meetings were considered helpful. A culture of openness and mutual appreciation between research and practice in general was also described as beneficial. Familiarity with colleagues and residents, as well as long-term integration in the care team were considered helpful for the PLP role.

Concerning the Individuals domain, implementation was influenced by roles at multiple levels. Barriers were sometimes a lack of support from senior- and mid-level leaders, e.g. through low prioritisation and limited involvement. Unfamiliar (research) tasks, such as conducting a survey and giving presentations, led to uncertainty. Unclear role descriptions, role boundaries and the feeling of being solely responsible for project success increased uncertainties for the LPs. Differences in working styles and approaches between research and practice led to misunderstandings rooted in communication challenges of varying intensity across sites, particularly during the early stages. One SLP emphasised the need to build relationships early on in order to establish a common working approach: *“You really have to prioritise these interpersonal relationships. If you put them on hold*,* I think you’re making things unnecessarily difficult for yourself.”* (SLP). Key promoting factors were strong intrinsic motivation, enthusiasm for innovation and commitment to the topic of dementia. An academic background seemed to promote greater independence and self-efficacy in the case of one PLP who held a bachelor’s degree in nursing. Continuous competences growth strengthened the confidence of the LPs in their roles. Communication skills, practical experience and the ability to coordinate structures and integrate diverse perspectives were considered valuable. In addition, sufficient ‘time on site’ and integration into a broad network (including international exchange) were encouraging.

Finally, in the Implementation Process several facilitators and barriers were revealed. A lack of reflection opportunities, for example on new nursing interventions, was identified as a barrier. Participatory approaches, context-specific adaptations and systematic needs assessment on the level of the care facilities were identified as key success factors. Staff benefited from clearly structured, practice-oriented, easily integrable tasks and continuous communication. It also became apparent that interprofessional exchange and the inclusion of different perspectives can contribute to the sustainable establishment of Living Lab structures.

In our interviews, the LPs reflected on what they perceived as barriers and facilitators in adopting and carrying out their respective roles. Given the central role of the Linking Pins within the Living Lab, specific barriers and facilitators to adopting and executing the role were identified and summarised in addition to the CFIR-based contextual analysis (Table 6).

**Table 6:** Barriers and facilitators for linking pin roles

	PLP	SLP
Structural aspects	× Lack of a shared start to the LP-day × High workload, parallel projects, distraction by daily tasks × Insufficient equipment (technology, rooms) ✓ Joint start of all tasks, close support/supervision of SLP ✓ Well-structured meetings prepared by the SLP ✓ Fixed weekday for LP tasks ✓ Sufficient time off for project activities	× Lack of a shared start to the LP-day × Long travel times to the partnering facility (up to four hours) × Time challenges due to the dual role × High workload and unexpected challenges in practice ✓ Joint daily and overall planning ✓ Shared working mode ✓ Familiarity with relevant literature and reflection of the Living Lab structures
Individual aspects	× Uncertainties with project tasks (e.g. developing a questionnaire and conducting a survey or giving presentations) × Language barriers (Academic and English literature, scientific terminology) × Explaining project necessity to staff ✓ Long-term integration in the institution, practical experience and knowledge, positive attitude and perspective, motivation, ability to integrate other perspectives, organizational and reflective skills ✓ Position that combines practice and management fosters staff involvement ✓ Academic degree increased independence and personal responsibility	× High expectations for the SLP role and great responsibility regarding project progress × Immersion in the practical world: depending on the personality “type”; understanding language and codes in practice setting × Lack of decision-making ability (methodological uncertainties, hierarchical position) requiring high need for consultation with academic leads and other interest-holder ✓ Openness and communication skills ✓ Willingness to address prejudices towards science ✓ Build own relationships with institutions ✓ Experience and age
Aspects of collaboration and communication	× Different working methods and changes in SLP position; changes in internal roles × Limited exchange with nursing management and other interest-holders leads to isolation in the role × Switching between individual and team work ✓ Integration and acceptance in the PraWiDem team; support from the care team ✓ Exchange with and autonomy from management, exchange with senior researchers, other PLPs, international exchange and cross-location exchange ✓ Good relationship/good working atmosphere and support from the SLP; regular feedback from the SLP ✓ Detailed early-stage project discussions increased acceptance and justifiability of the project (of PLP)	× Different working methods between science and practice × Limited exchange between Living Lab locations × Unclear communication of expectations ✓ Institutional affiliation, intensive local exchange at the own Living Lab location, with PLPs and other SLPs, international exchange ✓ Dual role as a great opportunity for new insights and contribution ✓ Acceptance in the facility, “arrival” in the practical world, relationship work, also with staff, residents ✓ Collaboration with two PLPs ✓ Well-connected PLPs helpful as partner

*LPs* Linking Pins, *PLPs* Practice-based Linking Pins, *SLPs* Scientific Linking Pins

× stands for a barrier, ✓ for a facilitator

## Discussion

This study provides the first systematic evaluation of the degree of implementation, the mechanisms of impact, and the implementation barriers and facilitators of a Living Lab in healthcare. Our analysis revealed that the implementation of a Living Lab in German long-term dementia care was feasible, but the degree of implementation varied between clusters and intervention components. While the LP dyads were mostly implemented as planned, dose and reach of the PraWiDem team component were limited. The involvement of residents or relatives from the participating care facilities remained low. Co-creative research projects with staff were feasible and a central mechanism for knowledge circulation within the Living Lab. Facilitators for the implementation of the Living Lab ranged from interpersonal relationships to structured LP appointments and support from managers and senior researchers. Implementation barriers related among others to staff turnover, a lack of resources and initial role uncertainty among the LPs.

Our findings indicate that progress within the Living Lab relies heavily on the SLPs, especially in the beginning of the collaboration. The SLPs reported feeling primarily responsible for the establishment of the collaboration structures, initiating and supervising work on the research projects, and instructing PLPs and other staff. The variety of responsibilities was perceived as highly demanding. In addition, both SLPs and PLPs reported that their tasks were only outlined initially, but not specified in much detail, which means that the LPs may have been insufficiently prepared for their role. Inexperienced SLPs in particular reported initial role uncertainty and required considerable support from senior researchers. This finding is consistent with a previous report on the SLP role [29] indicating initial doubts and insecurities. Within the Limburg Living Lab, for example, the SLP role is usually assigned to experienced postdoctoral researchers [11]; other academic-practice partnerships have set similar requirements [30, 31]. While the SLPs in our Living Lab gradually settled into their roles over time, our results suggest that the skills of an advanced researcher are needed for the role. Based on our findings, we recommend that future SLPs should (i) possess research experience and qualification, (ii) be equipped with teaching skills and a broad range of participatory research methods to involve all interest-holder groups and (iii) have regular opportunities for role reflection with other experienced SLPs.

In accordance with our recruitment criteria, all PLPs were employed in direct patient care, which proved to be an important resource for involving other care professionals. However, only one PLP held a bachelor’s degree in nursing science. This is not surprising considering that there are only few academic nurses in direct patient care in Germany [32], and the number of bachelor degree graduates is particularly low in long-term care [33]. While our results suggest that not all LP tasks necessitate academic training (see Table 4), most LP tasks at the level of the research projects require some research-specific knowledge. Consequently, the PLPs needed extensive support to take on these tasks. Although conducting joint research projects may require a certain amount of guidance from the SLPs, this guidance cannot replace academic training, and contact time during the weekly LP appointments is too limited to acquire comprehensive research skills. Given that the bachelor’s degree of one of our PLPs proved to be highly valuable for working on the research projects, we conclude that recruiting PLPs with academic training may facilitate a common basis for LP collaboration on these projects. As the PLP role offers the opportunity to gain first-hand research experience in a familiar practice setting, it may be particularly attractive to nurses seeking to advance their academic training, e.g. at bachelor’s or master’s level. Moreover, it might be suitable for nurses seeking to combine their clinical practice with research activities, such as Advanced Practice Nurses (APNs). A recent review emphasises the role of APNs in evidence-based practice, but suggests that generating and synthesising evidence are the weakest competency areas of APNs [34]. Assuming the PLP role could therefore provide an opportunity to strengthen APNs’ research skills by enabling them to conduct joint research projects with experienced researchers. Overall, our results suggest that both SLPs and PLPs need thorough preparation for their roles and tasks before implementing a Living Lab. Further research may focus on the role-specific requirements and on developing training programmes and mentorship for the LP roles.

The implementation of the PraWiDem team component proved to be challenging. Based on our consultation with interest-holders during the adaptation of the Living Lab approach [15], we drafted the PraWiDem team component with the intention of creating tangible involvement opportunities for staff, residents and relatives. While our results mirror previous findings on academic-practice partnerships emphasising the importance of interpersonal relationships and involvement opportunities [35, 36], the involvement of staff in Living Lab activities was not limited to the PraWiDem team members. In addition, our recruitment efforts suggest that not all interested individuals were able or willing to take on the PraWiDem team role, e.g. due to inability to attend the monthly meetings. We therefore conclude that a designated team can be a valuable involvement opportunity in a Living Lab, but is not the only option for interest-holder involvement. Accessible participatory formats may be useful in involving different interest-holders who do not wish to make a long-term commitment to a specific role.

While the involvement of residents, for instance in the PraWiDem teams, was not achieved due to barriers such as advanced dementia, the recurring, moderated online consultations with the ‘Dementia and Research’ working group were both feasible and well-received by the working group members. Recent research suggests that homogenous working groups with dementia-specific adaptations (e.g. providing materials and questions in advance, moderation by trained facilitators) may be well-suited for involving people with dementia as such groups enable continuous involvement and a bidirectional flow of information [37]. This also better aligns with the key principles of patient and public involvement than one-time consultations with often a one-sided flow of information [37, 38]. However, given that our results suggest that initiating involvement opportunities within a Living Lab may be a responsibility of the LPs, we suggest that training on involvement methods should be considered as part of the LP role preparation in future Living Labs.

The joint research projects proved to be central to the Living Lab, as it was at the level of these projects that co-creation became tangible for all participants. Staff identification with the research projects was high in all clusters, and collaboration on the projects resulted in knowledge circulation through various formats. According to our previous consultation with interest-holder, many care professionals perceived research to be mostly inaccessible to them [15]. To counteract this impression, the research projects were carried out with the involvement of staff at every stage. This resulted in a high degree of co-creation and high acceptance, but may have led to limited pace, scope and scientific rigour of the projects. It is important to consider that (if not externally funded) the scope of the research projects conducted within the Living Lab depends heavily on the available resources. In our case, weekly LP appointments over an 18-month period amounted to 66 days (i.e. three months full-time employment). We interpret the co-creative research projects as an indication for the feasibility of our adapted Living Lab approach. However, for future Living Labs, it may be necessary to rebalance the degree of co-creation with the scope, rigour and available resources of the research projects, as well as the LP qualifications.

The barriers and facilitators identified in this process evaluation overlap considerably with previous findings on contextual factors influencing collaboration in academic-practice partnerships [39] as well as factors influencing the implementation of evidence-based practice [40, 41]. The CFIR-based analysis highlights aspects such as management and leadership support, team communication, time and other resources. Additionally, the identified aspects on the individual domain (CFIR) and the barriers and facilitators for the LP roles align with previously described personal factors, such as attitudes, confidence and meaningful recognition, and underscore the importance of personal relationships for collaborative partnerships [39]. This overlap highlights the importance of contextual factors for the implementation a Living Lab. The available AACN resources on the implementation of academic-practice partnerships [42] might help researchers and care organisations address implementation challenges if they are trying to collaborate in a Living Lab. Moreover, the international Living Lab collaboration might compare implementation barriers and facilitators across several countries in order to develop recommendations specifically aimed at the implementation of Living Labs. Building on our CFIR-based analysis of the contextual factors, the Expert Recommendations for Implementing Change (ERIC) would be a valuable addition to derive tailored implementation strategies [43].

### Strengths and limitations

The process evaluation was carefully planned and conducted in accordance with the UK MRC framework for the development and evaluation of complex interventions [14]. During the adaptation of the Living Lab approach, we developed a programme theory and visualised key assumptions on the implementation, mechanisms of impact, and contextual factors in a logic model, which guided the data collection for this study. As recommended, we chose a mixed-methods approach [14] and included several target groups in our evaluation [19], which provided a valuable diversity of perspectives on the Living Lab Dementia. All findings were cross-checked and repeatedly reviewed by experienced researchers to ensure the quality of our analyses.

However, our study also has some limitations. The participating care facilities were recruited through the researchers’ professional networks, and some of them had previously participated in other research projects. As a result, the sample may have comprised institutions with a higher affinity for research compared to care facilities in general. In view of the dual role of some SLPs who were also part of the research team, we excluded the research team as an intervention component from data collections on the mechanisms of impact and implementation context. This meant that the research team’s responsibilities relating to structural coordination and communication at the management level were not investigated, even though they may be crucial for setting up and maintaining a Living Lab. The exclusion of the research team probably influenced the richness of our data. Similarly, dual roles meant that the SLPs needed to analyse their own LP protocols. To reduce the risk of bias in these analyses, the findings were therefore discussed with researchers who held no SLP roles in a cross-cluster exchange. Furthermore, most research projects were still ongoing at the time of data collection. This may have influenced some of our findings, such as the PraWiDem team members’ perception of the impact of the Living Lab on dementia care in their facility. Lastly, we acknowledge that the joint research projects received considerable attention in this study. However, our process evaluation did not clarify whether the establishment of collaborative Living Lab structures represents a separate level of action for the intervention components (as indicated in our logic model), or whether collaboration on the research projects results in the establishment of collaborative structures. The development of these collaborative structures is not fully understood yet and may require further investigation.

## Conclusion

This process evaluation provides insight into the implementation of a Living Lab as an academic-practice partnership in long-term care. Despite implementation challenges arising from the demanding LP roles, staff shortages and limited reach of the PraWiDem team component, the implementation of our adapted Living Lab approach was feasible. Our findings indicate that LP roles and co-creative research projects may be important mechanisms for engaging various target groups in joint research within a Living Lab, and contribute to knowledge circulation between researchers, care professionals and people with dementia.

Our findings have several implications for future research. Firstly, the LPs were central within the Living Lab, but their roles proved to be more demanding than anticipated. Future studies might further explore the requirements for these roles and focus on developing guidance for role preparation or educational programmes for the LPs. Our findings on the barriers and facilitators for role adoption may serve as a starting point. Secondly, this study focused on the implementation of the adapted Living Lab approach and understanding its mechanisms of impact, but did not investigate Living Lab outcomes. In accordance with the UK MRC framework, we suggest that future evaluation studies focus on effects and outcomes and further refine our programme theory of the Living Lab approach. Additionally, future evaluations should investigate economic outcomes, which were not assessed in the present study since the grant-funded PraWiDem project did not incur costs for participating partners. Lastly, we acknowledge that, given the implementation of any complex intervention is highly context-dependent, our findings primarily reflect the implementation of a Living Lab in the context of German long-term care. Even though further studies are needed to understand how the Living Lab approach may be implemented into different care settings and how implementation contexts vary across countries, we believe that our findings provide valuable knowledge to understanding the change mechanisms within Living Labs. They lay a foundation for future implementation efforts of the Living Lab approach and offer tangible insights to support the translation of research into nursing practice.

Finally, we would like to note that, at the end of the PraWiDem study’s funding period, all participating long-term care organisations expressed their intention to continue the Living Lab and expand it to additional care facilities within their organisation. Consequently, the Living Lab has been adapted based on the findings of our study and is supported by intramural funding. The sustained Living Lab called PraWiLab is also broadening its focus to address a wider range of care needs in old age and will further develop the Living Lab approach in collaboration with the other European Living Labs [13, 44, 45].

## Supplementary Information

Supplementary Material 1.

## Data Availability

The datasets generated and analysed in this process evaluation are available from the corresponding authors upon reasonable request.

## Abbreviations

AACN: American Association of Colleges of Nursing
APN: Advanced Practice Nurse
CEO: Chief Executive Officer
CFIR: Consolidated Framework for Implementation Research
ERIC: Expert Recommendations for Implementing Change
LP: Linking Pin (Central role within the Living Lab. The two LPs work together as dyads for one day per week and are responsible for the establishment of joint collaboration structures as well as the identification, planning, conduct and dissemination joint research projects; for details on the LP tasks see Figure 1)
LTC: Long-term care
MRC: Medical Research Council
n.a.: not applicable
PLP: Practice-based Linking Pin (Care professional employed at a long-term care organisation within the Living Lab, who has taken on the LP role, collaborates with the Scientific LP for one day per week)
PraWiDem: German acronym for linking professional nursing practice and research in dementia
SLP: Scientific Linking Pin (Researcher employed at a university-based research institute within the Living Lab, who has taken on the LP role, collaborates with the Practice-based LP for one day per week)
