# Supplementary material for: Living Lab Dementia: Mixed-methods process evaluation of a feasibility study of an academic-practice partnership in German long-term dementia care
Source: BMC Geriatr. 2026 Feb 20;26:419. doi: 10.1186/s12877-026-07164-9 (PMC13032344; doi:10.1186/s12877-026-07164-9)
Supplement: Supplementary file 1 — Supplementary Material 1. [file 12877_2026_7164_MOESM1_ESM.docx]

# Supplements

## Supplement 1: Main questions of target group-specific interview guidelines

Individual interviews with Practice-based Linking Pins (PLP)

- Please start by telling me your overall impression of your two years in the Living Lab. How did you experience it?
- What was your day-to-day work like as a LP?
  - How did you plan and evaluate activities during the LP days?
  - What kind of support did you need for your work?
  - What were the opportunities for people with dementia to get involved with the Living Lab?
- What was important for you to be able to take on the LP role?
  - Which skills did you need?
  - How motivated were you throughout the collaboration?
  - How did you experience your collaboration with the Scientific LP?
  - In your opinion, what significance do the research projects have for the Living Lab?
  - How would you rate your personal increase in knowledge as a result of the research project?
- In your opinion, which external factors have influenced the collaboration in the Living Lab?
  - Which external factors were barriers, which were facilitators?
- Do you have any recommendations for future Living Lab projects in long-term care based on your experience in this project?
- Is there anything else you want to discuss?

Individual interviews with Scientific Linking Pins (SLP)

- Please start by telling me your overall impression of your two years in the Living Lab. How did you experience it?
- What was your day-to-day work like as a LP?
  - How did you plan and evaluate activities during the LP days?
  - What kind of support did you need for your work?
  - What were the opportunities for people with dementia to get involved with the Living Lab?
- What was important for you to be able to take on the LP role?
  - Which skills did you need?
  - How motivated were you throughout the collaboration?
  - How did you experience the overall communication during the collaboration in the Living Lab (other LPs, PraWiDem team, research team, international)?
  - In your opinion, what significance do the research projects have for the Living Lab?
  - How would you rate your personal increase in knowledge as a result of the research project?
- In your opinion, which external factors have influenced the collaboration in the Living Lab?
  - Which external factors were barriers, which were facilitators?
- Do you have any recommendations for future Living Lab projects in long-term care based on your experience in this project?
- Is there anything else you want to discuss?

Focus groups with PraWiDem-Team members

- Please start by telling me your overall impression of your two years in the Living Lab. How did you experience it?
- What was your day-to-day work like as a PraWiDem-Team member?
  - How did you experience the overall communication during the collaboration in the Living Lab (LPs, PraWiDem team, research team, international)?
  - In your opinion, what significance do the research projects have for the Living Lab?
  - How would you rate your personal increase in knowledge as a result of the research project?
- In your opinion, which external factors have influenced the collaboration in the Living Lab?
  - Which external factors were barriers, which were facilitators?
- Do you have any recommendations for future Living Lab projects in long-term care based on your experience in this project?
- Is there anything else you want to discuss?

Focus group with nursing management staff

- Please start by telling me your overall impression of your two years in the Living Lab. How did you experience it?
- What motivated you to participate in the Living Lab with your facility?
- How did you experience your role in the Living Lab?
  - How did you experience the overall communication during the collaboration in the Living Lab (LPs, PraWiDem team, research team, international)?
  - In your opinion, what significance do the research projects have for the Living Lab?
- In your opinion, which external factors have influenced the collaboration in the Living Lab?
  - Which external factors were barriers, which were facilitators?
- What do you expect from a long-term commitment to the Living Lab?
- Do you have any recommendations for future Living Lab projects in long-term care based on your experience in this project?
- Is there anything else you want to discuss?

Individual interviews with care organization CEOs

- Please start by telling me your overall impression of your two years in the Living Lab. How did you experience it?
- What motivated you to participate in the Living Lab with your facility?
- How did you experience your role in the Living Lab?
  - How did you experience the overall communication during the collaboration in the Living Lab (LPs, PraWiDem team, research team, international)?
- In your opinion, which external factors have influenced the collaboration in the Living Lab?
- Is there anything else you want to discuss?

Focus group with the working group ‘Dementia and Research’

- First of all, please tell us a little about your overall impression of two years of PraWiDem. How did you experience it?
- What opportunities did you have to take part in the PraWiDem project
  - How satisfied were you with these opportunities?
  - How did you experience the general exchange during the collaboration in the Living Lab?
- In your opinion, which external factors have influenced the collaboration within PraWiDem?
- Is there anything else you want to discuss?

## Supplement 2: Code system for qualitative data analysis

| 1. Implementation: reach |
| --- |
| - 1. Reasons for participation of care facilities |
| 1. Implementation: fidelity |
| - 1. Support requirements of Practice-based LP |
| - 1. Support requirements of Scientific LP |
| - 1. Project planning processes |
| 1. Implementation: adaptation |
| - 1. Adaptation processes |
| - 1. Reasons for adaptations |
| 1. Mechanisms of impact: LP dyad |
| - 1. Role identity Practice-based LP |
| - 1. Competencies Practice-based LP |
| - 1. Motivation and engagement Practice-based LP |
| - 1. Barriers for the realisation of the role as Practice-based LP |
| - 1. Facilitators for the realisation of the role as Practice-based LP |
| - 1. Role identity Scientific LP |
| - 1. Competencies Scientific LP |
| - 1. Motivation and engagement Scientific LP |
| - 1. Barriers for the realisation of the role as Scientific LP |
| - 1. Facilitators for the realisation of the role as Scientific LP |
| 1. Mechanisms of impact: PraWiDem team/circle |
| - 1. Acceptance and perception of the LPs |
| - 1. Competencies of PraWiDem team/circle members |
| - 1. Motivation and engagement of PraWiDem team/circle members |
| - 1. Expectations and management of expectations |
| 1. Mechanisms of impact: Management level |
| - 1. Acceptance and perception of the LPs |
| - 1. Competencies of LPs and PraWiDem team/circle |
| - 1. Expectations and management of expectations |
| 1. Mechanisms of impact: People with dementia and their relatives |
| - 1. Involvement opportunities |
| - 1. Satisfaction with involvement opportunities |
| 1. Mechanisms of impact: (Inter-)Professional relationships |
| - 1. Communication |
| - 1. Involvement |
| 1. Mechanisms of impact: Research projects |
| - 1. Co-creation |
| - 1. Consistency of realisation |
| - 1. Perceived knowledge circulation |
| - 1. Outcomes |
| - 1. Dissemination of outcomes |
| 1. Contextual factors: Implementation facilitators |
| - 1. Facilitators at micro-level |
| - 1. Facilitators at meso-level |
| - 1. Facilitators at macro-level |
| 1. Contextual factors: Implementation barriers |
| - 1. Barriers at micro-level |
| - 1. Barriers at meso-level |
| - 1. Barriers at macro-level |
